# Metabolic clustering of risk factors: evaluation of Triglyceride-glucose index (TyG index) for evaluation of insulin resistance

**DOI:** 10.1186/s13098-018-0376-8

**Published:** 2018-10-05

**Authors:** Sikandar Hayat Khan, Farah Sobia, Najmusaqib Khan Niazi, Syed Mohsin Manzoor, Nadeem Fazal, Fowad Ahmad

**Affiliations:** 1 Department of Pathology, PNS HAFEEZ, Islamabad, Pakistan; 2 CMH Multan, Lahore, Pakistan; 3 Healthcare Administration, Islamabad, Pakistan; 4 Department of Medicine, PNS HAFEEZ, Islamabad, Pakistan

## Abstract

**Background:**

Metabolic syndrome over the years have structured definitions to classify an individual with the disease. Literature review suggests insulin résistance is hallmark of these metabolic clustering. While measuring insulin resistance directly or indirectly remains technically difficult in general practice, along with multiple stability issues for insulin, various indirect measures have been suggested by authorities. Fasting triglycerides-glucose (TyG) index is one such marker, which is recently been suggested as a useful diagnostic marker to predict metabolic syndrome. However, limited data is available on the subject with almost no literature from our region on the subject.

**Objective:**

1. To correlate TyG index with insulin resistance, anthropometric indices, small dense LDLc, HbA1c and nephropathy. 2. To evaluate TyG index as a marker to diagnose metabolic syndrome in comparison to other available markers.

**Design-cross-sectional analysis:**

Place and duration of study-From Jun-2016 to July-2017 at PSS HAFEEZ hospital Islamabad.

**Subjects and methods:**

From a finally selected sample size of 227 male and female subjects we evaluated their anthropometric data, HbA1c, lipid profile including calculated sdLDLc, urine albumin creatinine raito(UACR) and insulin resistance (HOMAIR). TyG index was calculated using formula of Simental-Mendía LE et al. Aforementioned parameters were correlated with TyG index, differences between subjects with and without metabolic syndrome were calculated using Independent sample t-test. Finally ROC curve analysis was carried out to measure AUC for candidate parameters including TyG Index for comparison.

**Results:**

TyG index in comparison to other markers like fasting triglycerides, HOMAIR, HDLc and non-HDLc demonstrated higher positive linear correlation with BMI, atherogenic dyslipidemia (sdLDLc), nephropathy (UACR), HbA1c and insulin resistance. TyG index showed significant differences between various markers among subjects with and without metabolic syndrome as per IDF criteria. AUC (Area Under Curve) demonstrated highest AUC for TyG as [(0.764, 95% CI 0.700–0.828, p-value ≤ 0.001)] followed by fasting triglycerides [(0.724, 95% CI 0.656–0.791, p-value ≤ 0.001)], sdLDLc [(0.695, 95% CI 0.626–0.763, p-value ≤ 0.001)], fasting plasma glucose [(0.686, 95% CI 0.616–0.756, p-value ≤ 0.001)], Non-HDLc [(0.640, 95% CI 0.626–0.763, p-value ≤ 0.001)] and HOMAIR [(0.619, 95% CI 0.545–0.694, p-value ≤ 0.001)].

**Conclusion:**

TyG index, having the highest AUC in comparison to fasting glucose, triglycerides, sdLDLc, non-HDLc and HOMAIR can act as better marker for diagnosing metabolic syndrome.

## Background

Cardiovascular disease (CVD), non-alcohalic fatty liver disease (NAFLD)/Non-alcohalic Steato-hepatitis (NASH) and polycystic ovarian syndrome (PCOS) lead to enormous burden in morbidity and mortality along with effects on health economics. “Common soil hypothesis” suggests the singular entity to be responsible for most of these disorders, and that is resistance to insulin action [1]. Insulin resistance syndrome result in inability of insulin to exert their effects at target issues thus cause appearance of various abnormality spanning from NAFLD, NASH, PCOS to CVD [2]. Therefore, knowledge about insulin resistance in these subjects seems instrumental in identifying diagnosis and further on the management strategy [3]. Various direct and indirect measures to estimate insulin have been proposed starting from euglycemic clamp test to surrogate markers like QUICKI, HOMAIR and Matsuda Index [4]. However, in clinical practice at the primary care level it’s not only difficult to measure due to cost-effects but also stability of the insulin in blood becomes a question mark [5]. Thus the primary care physician needs a simple, robust and available marker as a surrogate for insulin resistance to address this very common pathology.

Recent evidence has suggested that calculated measure incorporating triglyceride and glucose, termed “Fasting triglyceride-glucose index” or simply “TyG index” has been suggested to help as surrogate marker for insulin resistance. Initial studies by Simental-Mendía et al. and Abbasi et al. [6, 7] have demonstrated their utility as a more tangible marker for metabolic syndrome and underlying insulin resistance. This measure only involves simple lab parameters like triglycerides and glucose, which can be measured without much effort or cost. Additionally, the parameter has even been shown to predict insulin resistance in a better manner than surrogate markers like HOMAIR once compared with direct measure like hyperglycemic clamp method [8]. Apart from metabolic syndrome, knowing its effect on nephropathy and atherogenic dyslipidemia will be interesting, as metabolic syndrome does not specifically include these parameters into its conventional definition. Moreover, relation between raised ALT levels and TyG index also needs to be evaluated, as Simental-Mendía [9] has also shown TyG index as a possible marker for diagnosing NASH among female subjects. Finally, multiple studies within our country have shown have evaluated metabolic syndrome and insulin resistance, the search on PakMediNet.com did not yield any study evaluating TyG index among our population cohort.

In the light of the promise as shown by few studies on TyG index to diagnose metabolic syndrome and its association with insulin resistance we decided to evaluate the performance of TyG index with insulin resistance, atherogenic dyslipidemia, anthropometric indices and urine albumin creatinine ratio. Furthermore, we also want to measure the performance of TyG index in diagnosing metabolic syndrome in comparison to insulin resistance and certain other anthropometric and lipid and non-lipid biomarkers.

## Methods

This comparative cross-sectional analysis was carried out from April-2016 to July-2017 at the department of pathology and department of medicine, PNS HAFEEZ Hospital (Islamabad, Pakistan). Formal approval was taken from the hospital ethical review committee regarding the study project before the start of study. Based upon non-probability convenience sampling and targeting patients who reported to us in “exact medical fasting status” we invited them to participate in the study. The subjects who volunteer were further evaluated for the presence of chronic disorders, exactness of medical fasting and taking anti-diabetic or anti-hypertensive drugs which could confound our results, pregnancy, any acute medical or surgical conditions and age < 18 years. Presence of these conditions implied exclusion from enrollment into study. Finally selected individuals (n = 228). Sample size was calculated based upon http://www.raosoft.com/samplesize.html.

Male subjects who had an initial raised level of total cholesterol were invited to the study. Subjects who had diabetes, hypertension, ischemic heart disease, age < 18 years or having any other chronic or acute ailments, taking any routine medication were excluded from the study. Those who initially consented verbally were requested to come to pathology department in “exact medical fasting status”. After a brief questionnaire based clinical history, subjects were evaluated for anthropometric indices and blood pressure. Anthropometric indices including height, weight, waist and hip circumference were calculated as per WHO criteria available at: http://www.who.int/childgrowth/publications/physical_status/en/. Following that blood specimens were collected in following tubes as Na-Fluoride, EDTA and plain bottles for evaluation of glucose, lipid profile, insulin, ALT and HbA1c. Urine specimen were collected in 171 subjects for urine albumin creatinine ratio (UACR) which is considered as a surrogate marker for nephropathy [10]. Glucose, total cholesterol, triglycerides and ALT were measured on Selectra-ProM random access clinical chemistry analyzer by following methods: GOD-PAP method, CHOD-PAP method, GPO-PAP, IFCC recommended kinetic method at 37°C. We measured serum HDLc and LDLc by cholesterol esterase method on AVIDA-1800 (Clinical chemistry system). HbA1c was analyzed by ion exchange resin chromatography, while insulin on serum was measured by chemiluminescence technique on Immulite^®^ 1000. AVIDA-1800 was also utilized to measure UACR. HOMAIR, TyG index and small density LDL-cholesterol (sdLDLc) were calculated vide given references [6, 11, 12].

We lost following samples during processing either due hemolysis or quantity was not sufficient for analysis as patient never appear for a re-test as: 2 for HDLc, LDLc and 4 for HbA1c and insulin.

### Data analysis

Patient data was initially entered into Excel work sheath and later moved to SPSS. Descriptive statistics for subjects were calculated using SPSS descriptive statistics. Parameters like waist to height ratio, BMI, waist to height ratio, fasting triglycerides, HDLc, TyG index, uric acid, non-HDLc and HbA1c were compared for gender differences by Independent sample t-test. Pearson correlation was used to correlate fasting triglycerides, HDLc, TyG and HOMAIR with various anthropometric indices and biochemical parameters. The differences among subjects having IDF-defined metabolic syndrome and otherwise for various end-points were measured using independent sample t-test. ROC curve analysis was utilized with presence or absence of metabolic syndrome as per IDF defined criteria to compare area under the curve (AUC) for candidate metabolic syndrome markers including fasting plasma glucose, triglycerides, non-HDLc, sdLDLc, HOMAIR and TyG index.

## Results

**Fig. 1:**
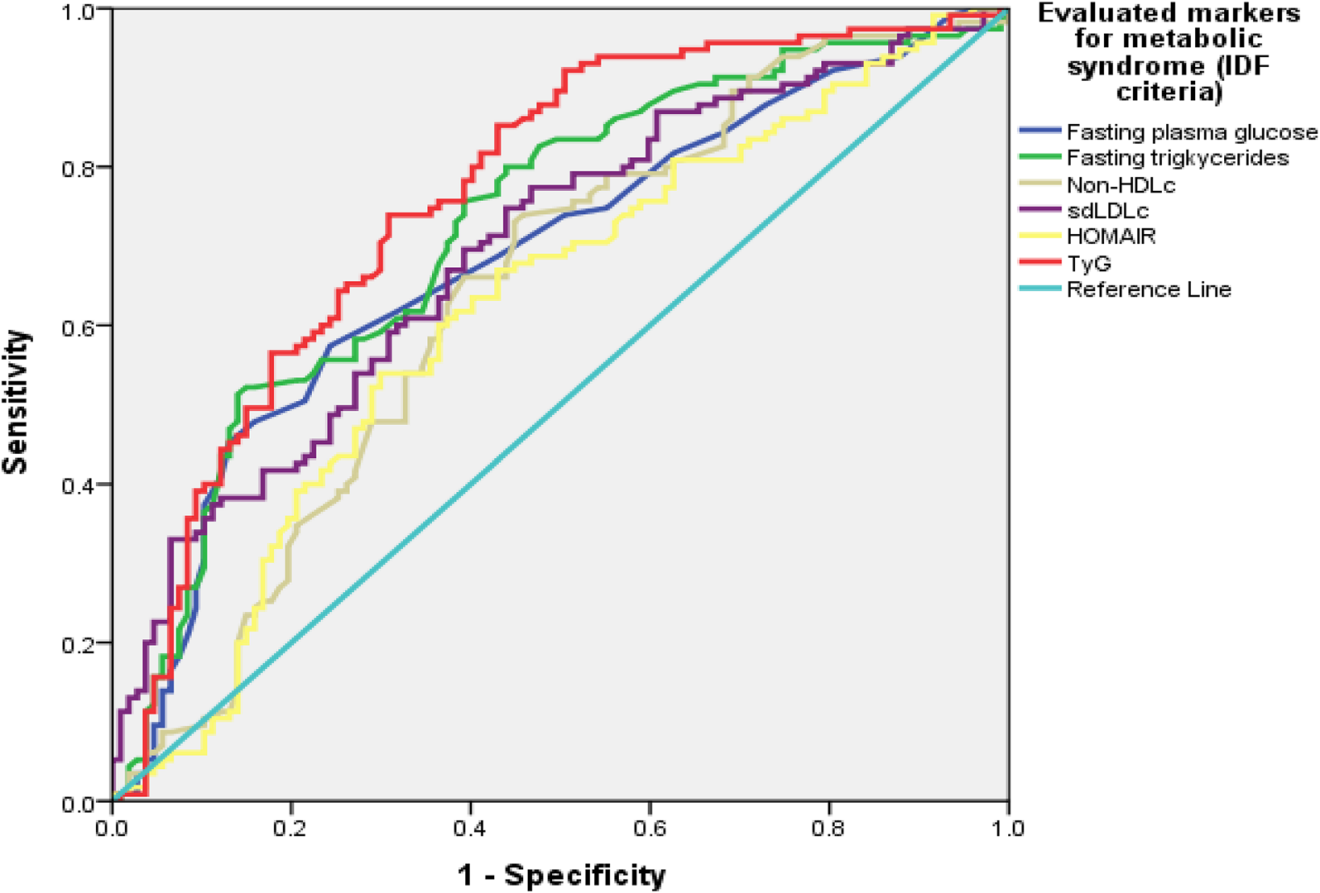
ROC curve analysis for predicting evaluated markers AUC against a diagnosis of metabolic syndrome as per IDF criteria. (n=227)

We had a total of 227 subjects in our sample with 118 females and rest males. Details of data on age, anthropometric and biochemical measures from our study population are shown in Table 1. Few parameters including BMI, waist to height ratio, fasting triglycerides, TyG index, HDLc, uric acid, non-HDLc, HbA1c were significantly different between male and female subjects (Table 2). TyG in comparison to other markers like fasting triglycerides, HOMAIR, HDLc and non-HDLc was the only marker which showed higher or near equivalent linear correlation with multiple BMI and other biochemical parameters including atherogenic dyslipidemia surrogate sdLDLC, nephropathy, glycated hemoglobin and insulin resistance as depicted in Table 3. Differences between various markers among subjects with and without metabolic syndrome as per IDF criteria are shown in Table 4. In order to evaluate diagnostic efficiency by measuring AUC (Area Under Curve) for TyG and other potential markers including fasting triglycerides, fasting plasma glucose, HOMAIR, sdLDLc and Non-HDLc we observed highest AUC for TyG as [(0.764, 95% CI 0.700–0.828, p-value ≤ 0.001)] followed by fasting triglycerides [(0.724, 95% CI 0.656–0.791, p-value ≤ 0.001)], sdLDLc [(0.695, 95% CI 0.626–0.763, p-value ≤ 0.001)], fasting plasma glucose [(0.686, 95% CI 0.616–0.756, p-value ≤ 0.001)], Non-HDLc [(0.640, 95% CI 0.626–0.763, p-value ≤ 0.001)] and HOMAIR [(0.619, 95% CI 0.545–0.694, p-value ≤ 0.001)] (Fig. 1).

**Table 1:** Descriptive statistics of age, anthropometric and biochemical measures in our data set

Parameters	N	Mean	Std. dev	Kurtosis	
				Statistic	Std. error
Age (years)	227	46.37	11.98	0.553	0.322
Body mass index (BMI)	227	27.10	5.24	1.970	0.322
Waist to hip ratio (WHpR)	227	0.93	0.08	70.77	0.322
Waist to height ratio (WHtR)	227	0.57	0.07	− 0.076	0.322
Fasting plasma glucose (mmol/L)	227	5.51	1.93	22.427	0.322
Total cholesterol (mmol/L)	227	4.47	0.61	0.563	0.322
Fasting triglycerides (mmol/L)	227	1.58	0.71	3.934	0.322
HDLc (mmol/L)	225	0.98	0.26	10.105	0.323
LDLc (mmol/L)	225	2.69	0.73	− 0.296	0.323
Non-HDLc (mmol/L)	227	3.50	0.64	0.492	0.322
Uric acid (mmol/L)	226	302.67	79.14	1.78	0.322
HbA1c (%)	223	5.73	0.93	3.04	0.324
HOMAIR	223	2.32	1.83	10.06	0.324
Urine albumin creatinine ratio	171	2.76	4.94	75.16	0.379

**Table 2:** Gender associated differences in age, anthropometric and biochemical measures in our sample population

Parameters	Gender	N	Mean	Std. Dev	Sig. (2-tailed)
Age (years)	Male	109	47.94	11.35	0.058
	Female	118	44.92	12.41	
Body mass index (BMI)	Male	109	25.96	4.85	0.002
	Female	118	28.15	5.39	
Waist to hip ratio (WHpR)	Male	109	0.93	0.10	0.223
	Female	118	0.94	0.06	
Waist to height ratio (WHtR)	Male	109	0.55	0.06	< 0.001
	Female	118	0.60	0.07	
Fasting plasma glucose (mmol/L)	Male	109	5.71	2.12	0.138
	Female	118	5.33	1.74	
Total cholesterol (mmol/L)	Male	109	4.54	0.59	0.111
	Female	118	4.41	0.61	
Fasting triglycerides (mmol/L)	Male	109	1.68	0.82	0.044
	Female	118	1.49	0.59	
Triglyceride-glucose index (TyG)	Male	109	8.80	0.58	0.030
	Female	118	8.65	0.46	
HDLc (mmol/L)	Male	108	0.91	0.21	< 0.001
	Female	117	1.04	0.28	
LDLc (mmol/L)	Male	107	2.71	0.68	0.624
	Female	118	2.66	0.77	
Non-HDLc (mmol/L)	Male	109	3.63	0.58	0.004
	Female	118	3.39	0.68	
Uric acid (mmol/L)	Male	108	330.36	81.61	< 0.001
	Female	118	277.33	67.79	
HbA1c (%)	Male	107	5.57	0.96	0.010
	Female	116	5.89	0.88	
HOMAIR	Male	107	2.28	1.96	0.707
	Female	116	2.37	1.70	
UACR	Male	75	2.31	2.47	0.292
	Female	96	3.11	6.21

**Table 3:** Correlation between fasting triglycerides, TyG, HDLc and HOMAIR with anthropometric indices and biochemical risks

Parameter		Fasting triglyceride(mmol/L)	(Glucose-triglyceride index) TyG	HDLc (mmol/L)	Non-HDLc (mmol/L)	HOMAIR
WHpR	Pearson correlation	0.154*	0.167*	0.002	0.178**	0.100
	Sig. (2-tailed)	0.020	0.012	0.974	0.007	0.136
	N	227	227	225	227	223
Fasting plasma glucose (mmol/L)	Pearson correlation	0.190**	0.571**	− 0.066	0.030	0.398**
	Sig. (2-tailed)	0.004	< 0.001	0.325	0.653	< 0.001
	N	227	227	225	227	223
Total cholesterol (mmol/L)	Pearson correlation	0.445**	0.385**	0.105	0.887**	− 0.013
	Sig. (2-tailed)	< 0.001	< 0.001	0.117	< 0.001	0.842
	N	227	227	225	227	223
TyG	Pearson correlation	0.869**	1	− 0.290**	0.460**	0.274**
	Sig. (2-tailed)	< 0.001	–	< 0.001	< 0.001	< 0.001
	N	227	227	225	227	223
LDLc (mmol/L)	Pearson correlation	0.003	0.069	− 0.005	0.381**	0.025
	Sig. (2-tailed)	0.970	0.302	0.941	< 0.001	0.715
	N	225	225	224	225	222
sdLDL (mmol/L)	Pearson correlation	0.540**	0.526**	− 0.133*	0.353**	0.085
	Sig. (2-tailed)	< 0.001	< 0.001	0.046	< 0.001	0.206
	N	227	227	225	227	223
HbA1c (%)	Pearson correlation	0.007	0.240**	0.058	− 0.087	0.193**
	Sig. (2-tailed)	0.915	< 0.001	0.387	0.784	0.004
	N	223	223	222	221	223
UACR	Pearson correlation	0.119	0.199**	− 0.080	0.100	− 0.017
	Sig. (2-tailed)	0.122	0.009	0.298	0.196	0.827
	N	171	171	170	170	171

*significant at  < 0.05

**significant at < 0.01

**Table 4:** Differences of various biomarkers in subjects with and without metabolic syndrome as per IDF criteria

Parameter	Metabolic syndrome	N	Mean	Std. dev	Sig. (2-tailed)
Fasting plasma glucose (mmol/L)	Present	117	5.72	1.85	0.070
	Absent	108	5.25	1.98	
Fasting triglyceride (mmol/L)	Present	117	1.79	0.68	< 0.001
	Absent	108	1.37	0.68	
Triglyceride-glucose index (TyG)	Present	117	8.91	0.42	< 0.001
	Absent	108	8.53	0.55	
HDLc (mmol/L)	Present	117	0.94	0.25	0.040
	Absent	108	1.02	0.26	
Non-HDLc (mmol/L)	Present	117	3.61	0.60	0.004
	Absent	108	3.36	0.65	
sdLDLc (mmol/L)	Present	117	0.92	0.33	< 0.001
	Absent	108	0.70	0.29	
HbA1c (%)	Present	115	5.93	0.94	0.001
	Absent	107	5.51	0.88	
HOMAIR	Present	115	2.53	1.77	0.084
	Absent	107	2.10	1.89

## Discussion

Our study highlighted that TyG index is the most efficient marker to diagnose metabolic syndrome. This finding is in accordance with the work of Simental-Mendía et al. and Abbasi et al. [6, 7]. Apart from the diagnosis of metabolic syndrome, we also observed a relatively better comparative linear correlation between TyG index and other cardiovascular (CVD) risk biomarkers for atherogenic dyslipidemia, nephropathy, underlying insulin resistance and hemoglobin glycation. The later finding further enhance the significance of TyG index as a “pan CVD risk marker”. The support from “TyG as a pan CVD marker” comes from studies carried out to link angiographic based risk findings in coronary artery disease (CAD) like obstructive CAD and coronary artery calcium scores were found to be more associated with TyG index and identified TyG as independent risk marker for CAD [13]. Similar studies from Korea have also identified the independent nature of TyG for depicting underlying cardiovascular diseases [14, 15]. Provided it’s independent nature as depicted by the aforementioned evidence shared, we could not find data linking TyG with small dense LDLc and nephropathy. So this area needs to be evaluated further.

Most data including our earlier work on the subject have shown HOMAIR as a very significant predictor of metabolic syndrome [16–18]. However, here we observed HOMAIR to show the least AUC in comparison to other evaluated markers. Probable explanations could be: Firstly, we believe that HOMAIR being the product of fasting insulin and glucose based upon a physiological mathematical modelling could depict risk related to insulin signaling pathway defects and may not be actually influencing risks resulting from hepatocyte function or alterations resulting from polygenic modes of causation of cardiovascular diseases [19, 20]. Secondly, HOMA model has inherent weakness and it may not be applicable to all patient groups like lean patients with metabolic risks, which is a common category in Asian population as highlighted by Kang et al. [21]. Available evidence review also suggests differential outcomes from two insulin resistance related measure HOMAIR, where the first version, the tradition one as utilized by us i.e., HOMA_1_ within our study and another one suggested by Levy et al. based upon a computer program being called HOMA_2_ [22, 23]. The latter version, by some authorities have been qualified as better version for Asian population [24]. However, we feel that more data on differences be two between the two equations is needed to validate our findings. Lastly, evidence from literature in certain specific patient groups suggests limited role of HOMAIR with associated cardiovascular disease mortality [25]. Similarly, evidence highlights that are racial and ethnic differences among human subjects with regards to insulin resistance, and type-2 diabetes mellitus [26–28].

We feel certain limitations to our study needs to be acknowledged: Firstly, our cross-sectional design in design which has inherent limitations. Furthermore, we feel more studies with a much larger sample size by clinical epidemiologists to conclude the real yield of this tests.

Provided the limitations we mentioned, we believe the study remains clinically valid as it provides a very simple mathematical marker for clinical use which is not only cost-effective but also be useful in small set ups with minimal laboratory facilities. Moreover, further augmentation of this biomarker any large-scale studies can help replace the varying definitions of metabolic syndrome which are sometimes creating diagnostic confusion and makes thing complex than easy in primary clinics.

## Conclusion

TyG index, having the highest AUC in comparison to fasting glucose, triglycerides, sdLDLc, non-HDLc and HOMAIR can act as better marker for diagnosing metabolic syndrome. Keeping in view the simplicity of marker, cost-effectiveness and feasibility at small-scale lab and being depictive of other cardiovascular risks it is suggested to incorporate this test in clinical use.

## Abbreviations

TyG index: triglyceride-glucose index
IDF: International Diabetic Federation
GPO-PAP: glucose peroxidase method
ROC: receiver operating curve
UACR: urine albumin creatinine ratio
sdLDLc: small dense LDL cholesterol
WHpR: waist to hip ratio
